# Triangle of Healthy Caregiving for Veterans With Spinal Cord Injury: Proposal for a Mixed Methods Study

**DOI:** 10.2196/14051

**Published:** 2020-05-12

**Authors:** Carol McMara Gibson-Gill, Joyce Williams, Denise Fyffe

**Affiliations:** 1 Veterans Administration New Jersey Health Care System East Orange, NJ United States; 2 Department of Physical Medicine and Rehabilitation Rutgers New Jersey Medical School Newark, NJ United States; 3 Kessler Foundation West Orange, NJ United States

**Keywords:** veterans health, spinal cord injury, telemedicine, telehealth, delivery of health care, virtual health, health services accessibility, quality of life, patient care team, caregivers

## Abstract

**Background:**

Spinal cord injury (SCI) is a debilitating injury that results in chronic paralysis, impaired functioning, and drastically altered quality of life (QOL). The Department of Veterans Affairs (VA) estimates that approximately 450 newly injured veterans and active-duty members receive rehabilitation at VA’s Spinal Cord Injury/Disorders Centers annually. VA virtual health services use technology and health informatics to provide veterans with better access and more effective care management. The “Triangle of Healthy Caregiving for SCI Veterans” is a patient-centered intervention that incorporates SCI veterans’ caregivers into the VA SCI health care team and extends into the homes of veterans with SCI by using real-time clinical video teleconferencing (CVT). CVT facilitates video-clinic visits, which can include different types of clinical evaluations, therapy (physical/occupational), or psychosocial services. The “Triangle of Healthy Caregiving for SCI Veterans” builds on interactive, interdisciplinary health care relationships that exist between the veterans with SCI, their caregivers, and the VA SCI health care team. SCI veterans’ propensity to multiple secondary complications makes a healthy partnership crucial for the success of keeping better health and functional outcomes as well as quality of life while living in their homes.

**Objective:**

The goal of the proposed mixed methods project will assess SCI veterans’, their caregivers’, and the VA health care team’s perspectives and experiences in the “Triangle of Healthy Caregiving for SCI Veterans” to determine the benefits, challenges, and outcomes for everyone involved in the intervention.

**Methods:**

Data collection methods will be implemented over three sequential phases. First, in-depth interviews will be conducted with the telehealth coordinators to systematically document the administrative procedures involved in enrollment of veterans with SCI into the CVT system. Next, structured observation of the CVT enrollment process and logistics of home installation of the CVT system will be conducted to validate the content of the in-depth interviews and highlight any discrepancies observed. Semistructured interviews will be conducted to assess specific elements of the “Triangle of Healthy Caregiving for SCI Veterans” program, their perceived utility, and effectiveness of the CVT system as well as the general impressions of the impact of the intervention on the SCI veterans’ health and function outcomes, caregiver burden, and daily caregiver burden. Finally, the research team will conduct a focus group to evaluate the ways in which the “Triangle of Healthy Caregiving for SCI Veterans” is useful for health care delivery to veterans with SCI and support services to SCI caregivers.

**Results:**

This proposal was funded in July 2017. It was reviewed and received institutional review board approval in March 2018, and the project was started immediately after, in the same month. As of September 2019, we have completed Phases I and III and have recruited 52 subjects for Phase II. We are beginning the data analysis. The study is projected to be completed in late summer of 2020, and the expected results are to be published in the fall of 2020.

**Conclusions:**

The findings from this study will highlight the ways in which virtual health care technologies can be used to improve access to SCI specialized care for veterans and provide an estimation of the potential impact on clinical outcomes for veterans with SCI and their caregivers.

**International Registered Report Identifier (IRRID):**

DERR1-10.2196/14051

## Introduction

### Background

Spinal cord injury (SCI) is a devastating and disabling medical condition with significant impact on quality of life (QOL) and finances on multiple levels for wounded members of the military, their families, and the health care system [1-3]. The Department of Veterans Affairs (VA) is the single largest comprehensive health care provider for SCI in the nation [4,5]. There are approximately 44,000 veterans with SCI receiving health care at VA facilities [4,5]. In addition to requiring specialized and costly clinical health care, SCI often results in physical limitations that make assistance from others critical to maintaining health and facilitating full societal integration [6,7]. As such, the role of caregivers is increasingly being recognized as instrumental to SCI health care management. Caregivers have been identified as critical members of the SCI medical rehabilitative team, who are responsible for providing assistance, supervision, and health care to persons living with an SCI, including veterans [8-13]. Despite the presence of a caregiver, accessing SCI specialty care may be further challenged with the development of complexities evident in chronic SCI, including the effects of aging, transportation costs, or the distance to the VA hospital or outpatient services [8-10,14-17]. Therefore, methods to facilitate improved access to rehabilitative medical care are crucial.

Virtual care is the practice of delivering health care to patients separated from the provider by a physical distance by using a variety of technologies [18]. It includes using land-based telephone communications as well as more advanced technologies as follows: (1) Telehealth, which uses technology that the patients use to enter health facts (eg, their blood pressure or fingerstick blood glucose level) that get transmitted to their provider, who can then make adjustments in the management of their condition based on this information. It also provides disease-specific education to the patients based on the patient’s responses to specific questions. (2) Telemedicine videoconferencing (clinical videoconference technology) permits real-time, secured, face-to-face visits between providers and patients. The patients are able to meet with individual providers or multiple members of the team simultaneously. Physical examination of the patients can be performed via some of these technologies as well as education, counseling, and other assessments. (3) Secure messaging (eg, veterans communicating securely with their health care team by email, which interfaces with their electronic medical record) [18]. All of these modalities have specific features to help veterans and their providers access each other in a timely fashion.

Over the past 16 years, the VA New Jersey Health Care System’s (VANJHCS’s) Spinal Cord Injury & Disorders (SCI/D) SCI health care team (HCT) has successfully implemented veteran-centered care for the SCI/D population. This veteran-centered care views veterans and their caregivers as one system that works in partnership with the HCT. Through this partnership, described as “The Triangle of Healthy Caregiving,” the HCT incorporates the use of virtual care technologies (real-time clinical video teleconferencing [CVT]) in their delivery of primary care and specialty services directly to the veteran and caregiver based on the clinical needs identified [19-21]. For the proposed project, the research team will use qualitative and quantitative data collection methods to assess the perspectives and experiences of key stakeholders involved in the intervention—veterans with SCI, their caregivers, and the VA health care team—to ascertain the benefits, challenges, and outcomes for these key stakeholders involved in the intervention.

### Research Problem

Improved health care access helps prevent costly secondary conditions among people with SCI [1,22-24]. Patients with spinal cord injury require lifelong monitoring to effectively address and prevent secondary conditions while promoting stability in functionality as they age [25]. The lifetime cost of providing care to patients with SCI can range from US $40,589 to US $177,808 annually, depending on the level of injury and age of initial injury [24]. In fiscal year 2004, the Veterans Health Administration SCI program accounted for approximately US $716 million in direct medical costs for 18,539 enrolled veterans (Veterans Health Administration intranet) [1]. A cross-sectional study using the National Spinal Cord Injury Statistical Center database reported that a significant proportion of persons with SCI visited a doctor for at least one medical complication at the time of their annual checkup, which does not include other medical conditions they did not have treated [26]. Utilizing telehealth technologies to provide ongoing primary and specialty health care to veterans with SCI is essential, as we seek to improve the experience of our veterans and their caregivers while reducing the cost of travel and the need for emergency care [16,25,27].

VA virtual health services use technology and health informatics to provide veterans with better access and more effective care management [27,28]. VA is improving patient-facing and clinician-facing electronic health systems by expanding the development and use of health-related virtual modalities. These modalities include telehealth; electronic consult, where the consultant makes recommendations to the referring provider based on patient chart reviews; Secure Messaging in MyHealtheVet, which allows veterans to access their medical records, order medication refills, and communicate with their VA health care team, etc, through this secured patient portal; and mobile apps. VA is aligning virtual care technologies to create a seamless, unified experience for all VA patient-facing technologies [27,28].

Despite advances in VA virtual care technologies and growing empirical evidence about the unique relationship between veterans with SCI and their caregivers, there is a growing need to fully understand how utilizing virtual care technologies impacts the health and QOL of both veterans with SCI and their caregivers [21]. Promising results from video/telecommunication technology studies with caregivers of veterans with SCI indicated improvements in the caregivers’ problem-solving skills and QOL outcomes [10,16,29,30]. To address the health care needs of veterans with SCI, support their caregivers, and provide timely access to health care providers, the VANJHCS’s SCI/D Department developed the “Triangle of Healthy Caregiving for SCI Veterans” [19-21]. The “Triangle of Healthy Caregiving for SCI Veterans” is a patient-centered program that incorporates SCI Veterans’ Caregivers into the VA SCI HCT and extends into the homes of Veterans with SCI using real-time CVT. CVT facilitates video-clinic visits, which include clinical evaluations, therapy (physical/occupational), and supportive services (eg, social work). The “Triangle of Healthy Caregiving for SCI Veterans” builds on interactive, interdisciplinary health care relationships that exist between the SCI veteran, their caregivers, and the VA SCI HCT. SCI veterans’ propensity to develop multiple secondary complications makes a healthy partnership crucial for the success of maintaining better health, functional outcomes, and overall QOL while the veterans live in their homes. Virtual medicine technologies can help improve accessibility by veterans with SCI and their caregivers to the VA SCI HCT in order to address issues in a timely fashion, reduce inconvenience or difficulties veterans with SCI may experience in reaching the health care facility to see their SCI specialists, and provide caregivers with timely educational and support services. The direct or indirect impact of the “Triangle of Healthy Caregiving for SCI Veterans” program has not yet been determined. The goal of this study is to assess the acceptability and utilization of the “Triangle of Healthy Caregiving for SCI Veterans” program by Veterans with SCI, Caregivers, and health care teams to modify, improve, and refine it accordingly.

### Specific Aims

This study aims to conduct a mixed methods descriptive study to assess the implementation process and outcomes of using CVT in the model of health care delivery that the SCI Center uses, called “Triangle of Healthy Caregiving for SCI Veterans.” Mixed methods research involves integrating quantitative and qualitative approaches to generating new knowledge. Combining methods activates their complementary strengths and helps overcome their discrete weaknesses [31]. Mixed methods have the advantage of allowing us to address these aims in a manner that is meaningful to those who are actively involved in the “Triangle of Healthy Caregiving for SCI Veterans”: veterans with SCI, family caregivers, and SCI clinicians.

#### Aim 1

Our first aim is to evaluate the SCI veterans’ experience in the “Triangle of Healthy Caregiving for SCI Veterans.” Our research questions are as follows:

1. What are SCI veterans’ perceptions about the provision and ways that the “Triangle of Healthy Caregiving for SCI Veterans” program impacts the delivery of health care?

2. What are the benefits and challenges the veterans with SCI experienced during implementation of the “Triangle of Healthy Caregiving for SCI Veterans” program in their homes?

#### Aim 2

Our second aim is to evaluate the SCI veteran caregivers’ experience in the “Triangle of Healthy Caregiving for SCI Veterans.” Our research questions are as follows:

1. What are SCI veteran caregivers’ perceptions about the “Triangle of Healthy Caregiving for SCI Veterans” program in management of caregiver burden?

2. What are the benefits and challenges that SCI veteran caregivers experienced during the implementation of the “Triangle of Healthy Caregiving for SCI Veterans” program in the homes of SCI veterans?

#### Aim 3

Our third aim is to evaluate the VA HCT’s experience in delivering health care and providing supportive services using the “Triangle of Healthy Caregiving for SCI Veterans.” Our research questions are as follows:

1. How do the “Triangle of Healthy Caregiving for SCI Veterans” health care professionals use the program to deliver care to Veterans with SCI?

2. Which elements of the “Triangle of Healthy Caregiving for SCI Veterans” work better in facilitating health care delivery? What are the key components of the program? Which components need to be revised?

## Methods

### Study Design

#### Preliminary Studies

As a result of clinical observations and anecdotal reports, the VANJHCS’s SCI Center’s Outpatient Clinic identified the financial, psychosocial, and other intangible costs specific to veterans with SCI, which incurred when they come to VANJHCS for care: (1) exorbitant cost of travel to/from appointments ranges from US $600 to US $2000 per visit, depending on the mode of transport (ie, wheelchair coach, stretcher, and advanced cardiovascular life support transport) and distance of veteran’s residence from medical center; (2) additional trauma to the wound during transport to and from the clinic appointment; (3) increased risk of developing more wounds during transport; and (4) inconvenience and discomfort for the veteran with SCI and caregiver.

Since the year 2000, the VANJHCS’ SCI Center has successfully integrated virtual care technologies to address negative factors associated with the travel to the VANJHCS SCI Center. The VANJHCS’s SCI/D SCI HCT implemented veteran-centered care for the SCI/D population. The “Triangle of Healthy Caregiving for SCI Veterans” utilizes a three-pronged approach to evaluations and educational interventions:

1. *Caregiving balance*: This component of the program focuses on educating veterans on what caregiving is from a caregivers’ perspective and the need for caregivers to care for themselves. Our veterans enlist in becoming caregiving partners with their caregivers, promoting a healthy relationship pattern.
2. *Caring for the caregivers:* This component educates and empowers Caregivers about ways to enhance the provision of care by learning to take care of themselves. Caregivers are also offered to participate in caregivers’ conferences where they learn from groups of caregivers and health care professionals about creative techniques to care for themselves. For example, caregivers learn coping techniques (eg, memoir writing, dance, and meditation) and skills they can implement on their own.
3. *VA virtual care HCT*: Veterans with SCI and caregivers have timely access to the SCI HCT using virtual care technologies to help prevent complications of medical issues seen after SCI as well as emotional and psychological burdens that would impact them and their caregiver’s capacity to provide the in home caregiving the veterans may need.

As a result of these key components, veterans with SCI report an increased understanding of what it means to be a caregiver to an SCI veteran. Our caregivers report a decreased sense of isolation and that they were implementing the coping skills they learned in their lives. Preliminary anecdotal feedback from the HCT indicate that veterans with SCI enrolled in the Home Telehealth Disease Management Protocols and the MyHealtheVet Secure Messaging platform have reported that the daily DMP sessions keep them focused on their health and wellbeing, and they learn new information through closer communications with their health care team. Veterans with SCI and caregivers reported that MyHealtheVet Secure Messaging is one of the best virtual care tools available because it is easy to use for renewing medications and messaging the SCI virtual HCT.

Key components and clinical procedures of the “Triangle of Healthy Caregiving for SCI Veterans" have been disseminated at professional conferences [19-36].

#### Participants

The veterans with SCI, SCI veteran caregivers, and virtual HCT will be recruited from the Spinal Cord Injury/Disorders (SCI/D) Department at the VANJHCS. The VANJHCS SCI/D Department serves an average of 480 SCI/D veterans on their patient registry with 147 veterans with SCI and 80 caregivers using the virtual care telehealth technologies.

### Data Collection

#### Subject Recruitment

The proposed study will receive approval from the VANJHCS Institutional Review Board. The study will collect data from three key stakeholders involved in the “Triangle of Healthy Caregiving for SCI Veterans” model of care who use CVT as part of the delivery of health care: veterans with SCI, SCI Veterans’ caregivers, and SCI virtual health care professionals (including telehealth coordinators). We will use purposive sampling to recruit a sample of veterans with SCI and caregivers who are newly referred and currently active or inactive users of CVT in the “Triangle of Healthy Caregiving for SCI Veterans” model of care. Purposeful sampling is a technique widely used in qualitative research that involves identifying participants that are especially knowledgeable about or experienced with a phenomenon [37].

Saturation is the point at which only minimal new information is gained from each new interview [38-40]. Data saturation has become the gold standard by which purposive sample sizes are determined in qualitative research [38-40]. The sample sizes proposed for each study phase described below are based on minimum sample size recommendations for common qualitative study designs [41]. Further, our sampling strategy will be flexible, evolving as the study progresses through the study phases until the point of redundancy in emerging themes is reached to meet the purposes of the study.

#### Veterans With Spinal Cord Injury

All veterans with SCI who receive clinical care at the VANJHCS are screened for enrollment on virtual care technologies as part of the “Triangle of Healthy Caregiving for SCI Veterans” program. However, based on the clinical experience of our research team, veterans with SCI at VANJHCS who are homebound, newly injured, affected by acute secondary complications (eg, pressure ulcer), and live in rural areas that are significant distance from the VANJHCS are more likely to enroll. There are currently 147 veterans with SCI actively using CVT in their homes. For the purposes of the study, we will recruit and enroll veterans with SCI based on the VANJHCS SCI/D clinical practice protocol. The inclusion criteria for the proposed study will include any veteran with SCI who is potentially or currently enrolled in the “Triangle of Healthy Caregiving for SCI Veterans” program. Veterans with SCI will be ineligible for entry into the study if any of the following exclusionary criteria are present: moderate to severe cognitive impairment or no ongoing landline or cell phone access.

#### Spinal Cord Injury Veterans' Caregivers

We will recruit caregivers of veterans with SCI at the VANJHCS who are potentially or currently enrolled in the “Triangle of Healthy Caregiving for SCI Veterans” program. There are currently 80 caregivers actively involved in the program. We will recruit a sample of 25-30 SCI veteran family caregivers who have provided care on a daily basis for at least 6 months to veterans with SCI and, preferably, these family members identify as the “primary” caregivers.

#### Telehealth Coordinators

We will conduct in-depth interviews with the two telehealth coordinators using an interview guide focused on existing structure and practices related to preparation and implementation of CVT in the homes of veterans with SCI who are enrolled in the “Triangle of Healthy Caregiving for SCI Veterans” program.

#### Spinal Cord Injury Virtual Care Clinical Team

Clinicians’ perceptions are important because they may affect patient-provider relationships, the course, and the outcome of treatment. Clinicians have knowledge of the medical and functional consequences of SCI and experience providing training to veterans with SCI and their family caregivers to plan for adjusting to home life and community reintegration. The SCI virtual health care team includes the following professional staff: physicians, advanced nurse practitioners, nurses, therapists (ie, occupational, physical, and recreation), social workers, psychologists, nutritionists, and clergy. We will recruit 8-10 VANJHCS SCI clinicians who are currently treating veterans with SCI and supporting their caregivers enrolled in the “Triangle of Healthy Caregiving for SCI Veterans” program to participate in one focus group.

#### Data Collection Methods

Data collection methods will be implemented over three phases of sequential qualitative and quantitative data collection outlined in Table 1. Results from each phase will be analyzed separately and then merged to inform the content of the subsequent phases as well as a set of recommendations for the “Triangle of Healthy Caregiving for SCI Veterans” to the VA National Office of Telehealth and the National SCI/D Systems of Care office.

**Table 1:** Data collection methods, purpose, and products.

Phase	Data collection methods	Purpose	Products
I	In-depth interviews	Conduct 2 in-depth interviews with the telehealth coordinators for the SCI^a^	Description of the enrollment and home installation process
	Observations of SCI Veterans’ enrollment and home installation	Conduct 15-20 observations of the patient enrollment and home installation of equipment/devices	Observation data of the enrollment and installation process
II	Semistructured interviews	Conduct 35-40 semistructured interviews with veterans with SCI enrolled in the “Triangle of Health Caregiving for SCI Veterans” program Conduct 25-30 semistructured interviews with caregivers of veterans with SCI enrolled in the “Triangle of Healthy Caregiving for SCI Veterans” program	Qualitative data (semistructured interview transcripts, field notes)
III	Focus groups	Conduct one focus group with virtual health care team professionals	Focus group findings about the delivery of health care to veterans with SCI, education, and support to their caregivers

^a^SCI: spinal cord injury.

#### Phase I: Enrollment and Installation of Equipment/Devices

We will use two qualitative data sources to assess the processes and logistics of enrollment in the program and the installation of CVT capability (equipment/devices/software) in SCI veterans’ homes: in-depth interviews and observations. In-depth interviews are one of the most common qualitative methods. In-depth interviews are open-ended interviews and enable respondents to discuss their point of view using their own language related to a topic with no predetermined list of responses. Structured observation of the CVT enrollment process and logistics of home installation of the CVT system will be conducted to validate the content of the in-depth interviews and highlight any discrepancies observed. Documentation data will consist of field notes that will be electronically recorded in Research Electronic Data Capture (REDCap) [42] (see Data Management System description below). The field notes will account for key events that took place during CVT enrollment and the home installation process and how the veteran with SCI or caregiver behaved or reacted in the interaction with the telehealth coordinator that services patients with SCI at VANJHCS.

### In-Depth Interviews With Telehealth Coordinators

We will conduct two in-depth interviews with telehealth coordinators to systematically document the administrative procedures involved in the enrollment of veterans with SCI into the CVT system. For example, the research team will ask administrative technicians to describe the ways in which description of CVT and various modalities and explanation of CVT installation/equipment in the home are discussed with veterans with SCI, and logistics of the delivery of CVT material and equipment will be reviewed. Additionally, the research team will assess the types of real-time problems of delivering health care to the veterans with SCI from the administrative technicians’ perspectives. They will summarize and review the information gathered from the interviews. The research team will use these data to develop observations forms to be used in the observations of the veterans’ enrollment and CVT equipment/device installation in their homes.

### Observations of Enrollments and Equipment/Device Installation

Direct observation will be performed of SCI veterans’ enrollment in the CVT program during consultations with the telehealth coordinators. The goal of this observation is to validate the information gathered from the in-depth interviews with the telehealth coordinators. Enrollment observations will record a face-to-face consultation with veterans with SCI (and caregiver) that was newly referred to the “Triangle of Healthy Caregiving for SCI Veterans” with the CVT administrative technician. The goal of the consultation is to provide veterans with SCI a description of CVT and various modalities, explanation of CVT installation/equipment in the home, and logistics of the delivery of CVT material and equipment and to obtain patient signatures (eg, commitment to ensure privacy during clinical visits, liability waiver, and protection of equipment the VA may give them).

Home observations of the installation process and utilization of the virtual care equipment in a sample of veterans with SCI households will provide the research team a context for the CVT installation process. The research team will take observation field notes to document the practicalities of CVT use in the home and any difficulties associated with home installation and usage. The observations will assess the length of installation time, the questions or concerns mentioned by the SCI veteran or caregiver during the installation, problems encountered by the CVT technicians during installation, and problems encountered during the testing of the CVT modalities (eg, accuracy of medical devices and display of educational modules).

#### Phase II: Semistructured Interviews

Semistructured interviews will be conducted to assess specific elements of the “Triangle of Healthy Caregiving for SCI Veterans” program, their perceived utility, and the effectiveness of the CVT system as well as general impressions of the impact of the intervention on the SCI veterans’ health and functioning outcomes (caregiver burden and daily caregiver burden). The semistructured interviews will include open-ended questions, closed-ended questions, and outcome measures. One-on-one interviews (in-person or via virtual care technology) with veterans with SCI (n=35-40) and their caregivers (n=25-30) will be conducted to capture SCI veterans’ and caregivers’ perceptions (including benefits and challenges) and experiences of participating in the “Triangle of Healthy Caregiving for SCI Veterans” program by using CVT. After consent is obtained, the research assistant will contact participants to complete a demographic questionnaire and health, function, and community participation outcome measures (Table 2). Upon completion of the outcome measures and semistructured interviews, participants will be compensated for their time.

**Table 2:** Outcome measures of veterans with spinal cord injury and caregivers.

Measures/scale		Outcome	Administration time (minutes)
**Veterans’ outcome measures**			
	Spinal Cord Injury Functional Index Short Forms (Jette et al 2012 [43]; Heineman et al 2014 [44])	Will ask persons with SCI^a^ to relate their perceived ability to complete function activities in four domains: wheel chair mobility, self-care, fine-motor function, and basic mobility	~5
	Veterans RAND 12-Item Health Survey (Selim et al 2008 [45])	Will assess quality of life thorough eight domains including physical functioning, vitality, role limitations due to physical problems, role limitations due to emotional problems, bodily pain, general health, social functioning, and mental health	<5
	Craig Handicap Assessment and Reporting Technique Short Form (Whiteneck 2011 [46])	Assess participation in society and subscales measuring physical independence, cognitive independence, mobility, occupation, social integration, and economic self-sufficiency	
**Caregivers’ outcome measures**			
	Caregiver Appraisal Scale (Lawton et al 1989 [47])	Measure caregiving satisfaction, perceived caregiving impact, caregiving mastery, caregiving ideology, and subjective caregiving burden	~15
	Caregiver Burden Scale (Elmstahl et al 1996 [48])	Assess amount of burden caregivers feel using five categories including general strain, isolation, disappointment, emotional involvement, and environmental strain	~10

^a^SCI: spinal cord injury.

A semistructured interview guide will be developed based on previous literature and findings of Phase I of this project. The interview will ask participants to express their perceptions and experiences with the “Triangle of Healthy Caregiving for SCI Veterans” program by using CVT. These interviews will give the research team the opportunity to further explore the topics that were of greatest interest and concern based on the observations in Phase I of the study. To ensure data quality, interviews will be audiotaped and transcribed. After the interview is completed, the research coordinator and research assistant will summarize their notes and review the results with the research team. Spot checks of the transcripts comparing them with the audiotapes will be performed to ensure accuracy of the transcripts.

#### Phase III: Spinal Cord Injury Virtual Medicine Clinician Focus Group

 We will conduct a focus group to evaluate the ways in which the “Triangle of Healthy Caregiving for SCI Veterans” is useful in health care delivery to veterans with SCI and support services to SCI caregivers. A sample of approximately 8-10 SCI virtual care clinicians will participate in 90-minute focus groups to derive a meaningful understanding of the ways in which CVT can provide health care to veterans with SCI and support to their caregivers. Focus groups capitalize on group interaction to produce data and insights that might be less accessible without interaction among individuals with common experiences [49,50]. The SCI virtual health care team includes the following professional staff: physicians, nurses, therapists (ie, occupational, physical, and recreation), nurse practitioners, social workers, psychologists, nutritionists, and clergy. The focus group discussion will ask clinicians to describe some of the key positive outcomes/results that have occurred in terms of patient care as a result of the introduction of the “Triangle of Healthy Caregiving for SCI Veterans” program and use of CVT (eg, cost savings, clinical effectiveness, and quality of life).

Focus groups are an efficient way to collect data from several people simultaneously, and they explicitly use group interaction as part of the method [49,50]. Focus groups will allow us to elucidate clinicians’ shared experiences and challenges of providing health care to veterans with SCI and support to veterans with SCI using the CVT technology in the “Triangle of Healthy Caregiving for SCI Veterans” program. The research coordinator or research assistant will take field notes on a structured data-recording sheet based on the focus group script/interview guide. The focus group will be recorded with a password-enabled digital recorder, and the recordings will be transferred to the secure VA network for transcription. The research team will debrief immediately after the focus group to share their impressions, critical points, and notable quotes.

#### Data Management System

We will utilize the VA Information Resource Center’s REDCap electronic data capture tool hosted by the VA Information Resource Center to store and manage the qualitative and quantitative data from each phase of the study [42]. REDCap is a secure Web app for building and managing online surveys and databases and permits data collection via a Web browser either locally or from remote locations. The NVivo (version 12; QSR International Pty Ltd, Melbourne, Australia) software supports mixed methods research to help research teams organize, analyze, and identify insights in unstructured or qualitative data** **and integrate quantitative data. NVivo also facilitates the export of demographic and qualitative data into quantitative analysis tools like SPSS (IBM Corp, Armonk, New York), which will be used for the quantitative analyses. The research team will integrate the qualitative and quantitative data.

#### Data Analysis

##### Qualitative Analyses

Qualitative data analyses will be guided by the Consolidated Framework for Implementation Research [51,52] using intervention-specific codes that will be developed throughout Phases I, II, and III by using a constant comparison analytic approach [53]. The research team will construct a preliminary codebook both deductively and inductively from the qualitative data and previous literature. Potential codes may include impact on patient health and functional outcomes, impact on health care utilization, reimbursement issues, CVT utility, and communication with health care team members. These codes will be applied using NVivo to develop an initial set of themes. The codebook will be elaborated upon and adjusted as the results of each phase of the study are reviewed, until thematic saturation is achieved within and across each phase of the study. Additional sources of qualitative data (eg, field notes) will be included in the dataset. The research team will summarize the data coded to the themes that will be independently reviewed by each member of the research team, discussed to derive consensus, and synthesized for each research question.

##### Quantitative Analyses

In addition to identifying themes and patterns qualitatively, we will examine the health and functional outcomes observed among veterans with SCI and caregivers in terms of the outcome data (aim 1 and 2) and coded qualitative data. We will explore descriptive data from Phases I to III using descriptive statistics (eg, means, SDs, percentiles, and ranges) and graphical techniques (eg, histograms and scatter plots) to characterize participant groups on key aspects (eg, working status).

Standard outcome measure scores will be generated by normative data. Separate analyses will be conducted for SCI veterans’ and caregivers’ outcome scores using analysis of covariance, with age and education as covariates. Once the qualitative data have been coded, more complex statistical analyses can be employed through the transformation of coded data into theoretically meaningful units of measure, as previously outlined [31,54,55]. This will allow examination of the differences between strategy utilization and health, function, and community participation outcomes.

#### Integrating Findings: Practice Recommendations

To design a useful set of practice recommendations, we will analyze results from each study phase separately and compare and merge the results across the quantitative and qualitative data sources. Qualitative and quantitative data will be triangulated. Triangulation is a methodological approach that contributes to the validity and reliability of integration when both qualitative and quantitative data collection methods are employed [56,57]. Triangulation will allow us to compare, contrast, and integrate the results from observations, interviews, focus group, and outcome measures. Triangulation from these three sources will also allow us to ensure the results are confirmed across data sources and identify data that are uniquely provided by different data sources. This is a side-by-side comparative analysis of the qualitative data and outcome scores to validate the findings across both sources of data collection.

Using the themes generated from the triangulated data, the research team will identify the most frequently cited factors (ie, benefits and challenges) that are important to key stakeholders in the “Triangle of Healthy Caregiving for SCI Veterans,” which are mentioned by more than one data source and across samples of participants—a process known as “group-to-group validation.” [50]. We will apply similar review methods to the differential patterns in the outcome measures. The research team will hold bimonthly consensus meetings to evaluate aspects of the most frequently cited benefits and challenges generated from the data based on the importance across the samples and modifiability of factors. After identifying factors that are both important and modifiable, the research team will prepare a summary of consumer-informed recommendations for the “Triangle of Healthy Caregiving for SCI Veterans” for the VA National Office of Telehealth and the national SCI/D Systems of Care office.

## Results

This proposal was funded in July 2017. It was reviewed and received institutional review board approval in March 2018, and the project was started immediately after, in the same month. As of September 2019, we have completed Phases I and III and have recruited 52 subjects for Phase II. We are beginning the data analysis. The study is projected to be completed in late summer of 2020, and the expected results are to be published in the fall of 2020.

## Discussion

SCI is a devastating, disabling medical condition with significant impact on quality of life and is very costly for patients, their families, and the health care system. Increasing access to specialized care can be paramount in preventing and managing the sequelae of SCI. Increasing the use and benefit of virtual care technologies in health care delivery have been noted, and the benefits of these technologies can also be seen when delivering care to people living with SCI. This study will help us understand the benefits, challenges, and outcomes of using virtual care technologies in health care delivery to veterans living with SCI by learning directly from veterans, their caregivers, and their health care team who have virtual health care integrated in the model of care. This information can also be expanded beyond the veteran population to potentially benefit all people living with SCI.

## Appendix

Multimedia Appendix 1 Peer-reviewer report.

## Abbreviations

CVT: clinical video teleconferencing
HCT: health care team
REDCap: Research Electronic Data Capture
SCI: spinal cord injury
SCI/D: Spinal Cord Injury/Disorders
QOL: quality of life
VA: Veterans Affairs
VANJHCS: Veterans Affairs New Jersey Health Care System
