# Supplementary material for: Triangle of Healthy Caregiving for Veterans With Spinal Cord Injury: Proposal for a Mixed Methods Study
Source: JMIR Res Protoc. 2020 May 12;9(5):e14051. doi: 10.2196/14051 (PMC7251480; doi:10.2196/14051)
Supplement: Multimedia Appendix 1 [file resprot_v9i5e14051_app1.pdf]

Department of Defense
U.S. Army Medical Research and Materiel Command
Congressionally Directed Medical Research Programs
2016 Spinal Cord Injury Research Program
Qualitative Research Award
Peer Review Summary Statement

CDMRP Log Number: SC160198
Grants.gov ID Number: GRANT12250720
Meeting Dates: 11/20/2016-11/21/2016
Review Panel: Qualitative Research

Project Duration: 36 months
Total Budget Requested: $527,245
Direct Costs: $443,721
Indirect Costs: $83,524

Title: Triangle of Healthy Caregiving for Spinal Cord–Injured Veterans
Principal Investigator: Carol Gibson-Gill
Performing Organization: Veterans Biomedical Research Institute, Inc.
Contracting Organization: Veterans Biomedical Research Institute, Inc.

Overview

The Principal Investigator (PI) of this application proposes to address the research problem of perspectives and experiences of spinal cord injury (SCI) veterans, their caregivers, and the Department of Veteran Affairs (VA) health care team in the "Triangle of Healthy Caregiving for SCI Veterans" to determine the benefits, challenges, and outcomes for everyone involved in the intervention, using a qualitative approach. The project's specific aims are to (1) evaluate SCI veterans' experiences in the Triangle of Healthy Caregiving for SCI Veterans, (2) evaluate the SCI veterans' caregivers' experience receiving support in the Triangle of Healthy Caregiving for SCI Veterans, and (3) evaluate the VA health care team's experience delivering health care and providing supportive services using the Triangle of Healthy Caregiving for SCI Veterans.

	Average Score	Standard Deviation
**Overall Evaluation**<br>Rating Scale: 1.0 (highest merit) to 5.0 (lowest merit)	1.8<br>**(Excellent)**	0.2
**Evaluation Criteria**<br>Rating Scale: 10.0 (highest merit) to 1.0 (lowest merit)	**Average Score**	
**Research Problem**	8.7	
**Study Design**	7.3	
**Impact**	8.4	
**Personnel**	7.8	

SCORED EVALUATION CRITERIA

Research Problem
Average Score: 8.7

Scientist Reviewer A

Strengths: The access to SCI specialty care is influenced by a number of factors, including aging and transportation costs or distance to the VA hospital or outpatient services. Therefore, methods to facilitate improved access to these services are critical. The proposed study is designed to increase the knowledge regarding how to improve and optimize the delivery of care via a virtual-care technology program, the Triangle of Healthy Caregiving for SCI Veterans. The investigation's goal, to best optimize this program, will be based on consumers', their caregivers', and the health care teams' perspectives and is an important and promising step to ensure better access to care, improve health outcomes for caregivers, and reduce costs of care for veterans with spinal cord injuries. The qualitative approach proposed is highly appropriate and critical to further understand the benefits, challenges, and outcomes for everyone involved in the intervention (eg, impact on the health and quality of life [QOL] of both SCI veterans and their caregivers, logistical issues). The rationale of the study is well supported, with the long-term goal of improving the health care delivery and quality of life for veterans with SCI. The application builds upon the VA New Jersey HealthCare System's SCI health care teams' experience with the "Triangle of Healthy Caregiving for SCI Veterans," which focuses on the caregiving balance, the caring for the caregivers, and the VA virtual care.

Weaknesses: No weaknesses were noted.

Scientist Reviewer B

Strengths: The research problem is well identified and supported. It is justified and fits the program announcement. The selection of qualitative methods is justified. It is well argued and supported by the rationale of the study and the research problem. This is a study that is based on previous health services research and the need to address a disparity in accessing rehabilitative medical care as a person with an SCI.

Weaknesses: No identifiable theoretical model for the study is stated or used to inform the research problem.

Biostatistician

Strengths: The Triangle of Healthy Caregiving for SCI Veterans is a program that seeks to marry tele-health care to the needs of people with SCI and their caregivers. The research project proposes to determine the impact of this intervention. This is a narrowly defined and well-designed study. Since the program is new, having an impact or effectiveness study would be a valuable addition. Qualitative research is very useful in these new areas and in environments of discovery. In this regard, the use of this method makes sense. Furthermore, the qualitative data are augmented by quantitative tools designed to evaluate functioning in participants, barriers to community life, and burden on caregivers. There is a strong value in understanding if this particular intervention will be useful to SCI patients and caregivers at some distance from a treatment setting.

Weaknesses: There is no discussion of theory found.

Procurement Sensitive Document
Do not copy or distribute without CDMRP written permission.

Discussion Notes

Reviewers agreed with the stated concern that a theoretical basis (theory) for the research is not noted.

Study Design
Average Score: 7.3

Scientist Reviewer A

The proposed study consists of 3 phases (ie, enrollment and installation, semistructured interviews, and SCI virtual medicine clinician focus group). Comparisons between direct observations of the veteran’s enrollment process and the in-depth interviews with tele-health coordinators will be made for validation purposes. Interviews will include open-ended questions, closed-ended questions, and outcome measures (in person or via virtual-care technology) with SCI veterans and their caregivers. This will be reviewed by the research team. SCI virtual medicine clinicians will participate in a 90-minute focus group.

Strengths: The proposed study will probe the perspectives of the principal stakeholders involved in the Triangle of Healthy Caregiving for SCI Veterans intervention (ie, SCI veterans, SCI veterans’ caregivers, and SCI virtual health care professionals). This interdisciplinary focus is likely to yield results that are critical for the optimization of the delivery of this model of care. The study design includes a rigorous analysis plan and data collection plan, including in-depth interviews, observations, and focus groups, that are likely to result in credible results. To ensure consistency and dependability of results, and prevent biases and preconceptions, the research team will construct a preliminary codebook based upon the qualitative data and on previous literature. These codes will be used to develop an initial set of themes. After this step, the codebook will be adjusted throughout the study until thematic saturation is achieved. The data will be independently reviewed by each member of the research team, discussed to derive consensus, and synthesized for each research question. In addition, triangulation will allow comparisons, contrast, and integration of the results from observations, interviews, the focus group, and outcome measures. The plan for recruitment appears sufficient. The SCI veterans, SCI veteran caregivers, and virtual health care team will be recruited from the Spinal Cord Injury/Disorders Department at the VA New Jersey Healthcare System. The inclusion criteria include any SCI veteran who is enrolled in the Triangle of Healthy Caregiving for SCI Veterans program (currently 147 SCI veterans). The proposed sample sizes are based on minimum sample size recommendations for common qualitative study designs but are somewhat flexible based upon saturation. Benchmarks are well described in the Statement of Work.

Weaknesses: There is no participant input on the list of codes to be developed. No potential problems and alternative approaches are mentioned.

Scientist Reviewer B

Strengths: The proposed design, sampling, and data collection are appropriate. The use of observations, in-depth interviews, and focus groups is appropriate; the number of participants is well justified. The applicant has explained rigorously how the team will develop the questions for the interviews. There do not appear to be any problems with access to or recruitment of the populations needed for this study. The applicant articulates in detail and with rigor the benchmarks to be utilized to stay timely and thorough.

Weaknesses: The draft questions provided for the veterans and caregivers are identical, and there is concern with the types of questions asked. Specifically, the first question is asking participants to identify change without a clear understanding of what that means. The third question seems to be directed to a

Procurement Sensitive Document
Do not copy or distribute without CDMRP written permission.

health care professional and not a service user. This is confusing. The PI doesn't give detail of how exactly the multiple data tools/methods, including the quantitative ones, will be combined or used to "triangulate."

Biostatistician

Strengths: The proposed project seeks to link advances in tele-health with a rich understanding of the impact of that technology on what is called the Triangle of Healthy Caregiving for SCI Veterans which recognizes the connections between healthcare providers, the individual with SCI, and the SCI patient's caregivers. In exploring how SCI patients experience the triangle of health care, researchers will interview both tele-health workers and virtual-care clinicians in order both to understand the triangle of healthy caregiving and to understand the ideal way that enrollment and care provision is designed to work. Observations are used to compare the descriptions of the enrollment process with the actual experience. Semistructured interviews are being used to understand the program participants' experiences with enrollment and with the delivery of care. Purposive sampling strategy is useful, particularly in new or recently developed areas of research. The number of tele-health workers seems reasonable to gather information on how the program is supposed to work. The focus group including 10 to 12 tele-health clinicians should create the possibility for a range of perspectives. The researchers are also using quantitative measures, as well, to measure health and functioning and community integration barriers that people with SCI are facing. They are also looking at the burdens faced by caregivers. The research team has direct access to the programs under study, providing a good opportunity to recruit the participants. The data analysis plan is strong, with its use of text management software and concern for maintaining credibility through the control of bias. This is accomplished in part by bringing on a senior qualitative researcher to help conduct and monitor the analysis. The researchers provide a detailed scope of work and set of milestones as well as steps they will use to achieve them.

Weaknesses: There is not a strong discussion of potential weaknesses for the study. Factors that go into an ideal participant are not included.

Impact
Average Score: 8.4

Scientist Reviewer A

Strengths: If successful, this study will result in an effective model of care that improves the access to SCI specialized care for veterans and their caregivers and improved QOL. A better understanding of the utility (including how to overcome barriers) of virtual-care technologies will facilitate the translation of this model of health care delivery to other SCI veterans. For those with SCI, an improvement of this health care model may reduce several of the current barriers to best care (eg, issues related to aging, transportation costs, or distance to the VA hospital or outpatient services) and thus improve satisfaction with care due to easier access and successful community integration. The study addresses a FY16 Spinal Cord Injury Research Program (SCIRP) Qualitative Research Award (QRA) area of encouragement (ie, identification and validation of best practices in SCI care).

Weaknesses: No weaknesses were noted.

Scientist Reviewer B

Strengths: This is a very important study, and its impact is clearly articulated and addressed in the narrative. It will assist in improving the QOL and delivery of care to persons with SCI and their

caregivers. This is a needed study, and it addresses a FY16 SCIRP QRA area of importance. The applicant justifies the need and impact of this study excellently.

Weaknesses: No weaknesses were noted.

Biostatistician

Strengths: If the project is successful, there will be a stronger understanding of whether the Triangle of Healthy Caregiving for SCI Veterans is an impactful method of improving the care received by patients with SCI and is useful in supporting caregivers. SCI patients face continuing health care challenges after leaving acute care. These challenges can last across the life course. Delivering high-quality care is an important goal, and this study seeks to understand the impact of this attempt to marshal tele-health resources in aid of this goal. The application responds directly to the quality of life after an SCI specified in the FY16 SCIRP QRA areas of encouragement. The study will illuminate how SCI patients view the use of this technology and the impact it has on their experience of receiving health care. There will also be insight into how the health care team views the use of this kind of technological mode of delivering health care.

Weaknesses: Attention to caregivers is too limited and may decrease the impact.

Consumer Reviewer

Strengths: The ability for a patient or caregiver to have direct access to his/her doctor or SCI team is advantageous. Being able to communicate and have the doctor see the issue and determine the next step without having to come to the clinic is a benefit. Not having to drive to the hospital will allow a person to continue their daily routine as well as help keep personal cost and time down to a minimum.

Weaknesses: The impact will be limited by individuals with SCI who do not have the ability to utilize this feature. There are already tele-health features established in some VA medical centers.

Discussion Notes

There was disagreement about the application’s impact, although generally it was considered moderate. Some reviewers agreed that the study would have high impact, while other reviewers agreed that the project’s decreased attention paid to caregiver issues relative to those of the individuals with SCI, given the central role caregivers frequently play in care for this population, slightly diminished enthusiasm.

Personnel
Average Score: 7.8

Scientist Reviewer A

The PI, Carol Gibson-Gill, received her MD in 1988 from Rutgers New Jersey Medical School, Newark, and completed a residency/fellowship in internal medicine/infectious diseases at Rutgers New Jersey Medical School, Newark, NJ, in 1994. Dr Gibson-Gill is the department chair of spinal cord injury/disorders at the VA New Jersey HealthCare. She is also the director of the New Jersey Region of the VA Multiple Sclerosis Center of Excellence-East and VA New Jersey’s director of the Amyotrophic Lateral Sclerosis Program.

Strengths: Dr Gibson-Gill is highly accomplished and appropriate as a PI for this application. She has earned multiple awards and has numerous years of experience with SCI patients. She initiated their department's caregivers program and continues to be actively involved in its use. Dr Gibson-Gill and her team have pioneered virtual care technologies in the service they provide to veterans and their caregivers and have over a decade of experience with this model of care. Dr Gibson-Gill has a program funded by Veterans Health Administration (VHA) Office of Rural Health "Seamless Care for Veterans with Spinal Cord Injury and Disorders," which is intended to improve access to specialty care by veterans across VISN lines. She is collaborating with the Kessler Institute on 2 Department of Defense–funded studies, "Systematic Assessment of Caregiving Skill Performance by Individuals with Tetraplegia and Their Caregivers" and "Spinal Cord Injury Veterans: Disability Benefits, Outcomes and Healthcare Utilization Patterns." Thus, she is very involved with projects highly relevant to the proposed study. The interdisciplinary research team members have different and complementary strengths that they bring to the proposed study. Joyce Williams is a licensed clinical social worker at the New Jersey VA and is the spinal cord injury and disorders coordinator for the NJ VA Healthcare System. She has expertise in psychosocial assessment, case management, veterans' and caregivers' support groups, and caregiver wellness conferences. She has extensive expertise in using virtual-care technologies. She collaborates on several grants relevant to this study. Denise Fyffe, PhD, is a senior research scientist at the Kessler Foundation with over 15 years of clinical research experience. Her background is in geriatric clinical psychology, specializing in home-based interventions with older adults. She has published expertise in qualitative methodologies and is currently a recipient of a research award from the Department of Defense to examine the impact of service-connected disability compensation on veterans with SCI in which she collaborates with Dr Gibson-Gill and Ms Williams. She lists a number of papers related to qualitative research, health disparities, and measurements and outcomes.

Weaknesses: Dr Gibson-Gill does not appear to list any/many peer-reviewed articles.

Scientist Reviewer B

Strengths: The research team has the training and experience to conduct the work proposed. The team's record of accomplishments is adequate.

Weaknesses: The qualitative key personnel level of effort is unclear, thus raising concern as to the successful conduct of the proposed work. The research analysis team is not described. This includes who will train the research assistants and how the team will resolve disagreements in coding and analysis.

Biostatistician

Strengths: The PI has assembled a very strong team to help conduct this study. The PI has a long history of expertise in the care of patients with SCI. She has also been an innovator in the domain of using tele-health and so is well positioned to explore the impact of the system under study. She has recruited an investigator from Kessler with a lengthy history of qualitative research and evaluation who brings high value to this project.

Weaknesses: No weaknesses were noted.

Discussion Notes

Reviewers agreed that the little or no reported peer-reviewed publication record of the PI diminished enthusiasm for the personnel.

Procurement Sensitive Document
Do not copy or distribute without CDMRP written permission.

UNSCORED EVALUATION CRITERIA

Environment

Scientist Reviewer A

The PI and her research team have access to the institutional/organizational resources at the New Jersey VA HealthCare as indicated by letters of institutional support. Additionally, there are letters from investigators Dr Fyffe and Ms Williams. As the chief of the VA SCI Service, Dr Gibson-Gill has access to the organization's infrastructure and subjects required to conduct the study successfully. The quality of the institutional/organizational support is deemed to be high and is appropriate for the proposed project.

Scientist Reviewer B

The scientific environment is appropriate. Availability and accessibility of resources is adequate.

Biostatistician

The scientific environment for this project is excellent. Both facilities are highly regarded. There should be little difficulty in recruiting the required numbers of participants among the patients, the tele-health implementers, or the health care providers. The research requirements can easily be met through both facilities. The institutional support will help the researchers to be successful.

Budget

Scientist Reviewer A

The budget is appropriate.

Scientist Reviewer B

The costs are very poorly calculated to allow for the level of work that is presented in this application.

Biostatistician

The budget seems appropriate for the study.

Application Presentation

Scientist Reviewer A

The application is clearly written and well presented. The application presentation did not influence the review.

Scientist Reviewer B

This is a well-written application. The application presentation did not influence the review.

Biostatistician

For the most part, the application is well presented and did not influence the review.

Procurement Sensitive Document
Do not copy or distribute without CDMRP written permission.
